# Landscape analysis for a neonatal disease progression model of bronchopulmonary dysplasia: Leveraging clinical trial experience and real-world data

**DOI:** 10.3389/fphar.2022.988974

**Published:** 2022-10-12

**Authors:** Jeffrey S. Barrett, Megan Cala Pane, Timothy Knab, William Roddy, Jack Beusmans, Eric Jordie, Kanwaljit Singh, Jonathan Michael Davis, Klaus Romero, Michael Padula, Bernard Thebaud, Mark Turner

**Affiliations:** ^1^ Critical Path Institute, Tucson, AZ, United States; ^2^ Metrum Research Group, Tariffville, CT, United States; ^3^ Tufts Medical Center and the Tufts Clinical and Translational Science Institute, Boston, MA, United States; ^4^ Division of Neonatology, Children’s Hospital of Philadelphia, Philadelphia, PA, United States; ^5^ Department of Pediatrics, Ottawa Hospital Research Institute, Ottawa, ON, Canada; ^6^ Department of Women’s and Children’s Health Institute of Translational Medicine, University of Liverpool, Liverpool, United Kingdom

**Keywords:** bronchopulmonar dysplasia, neonates, disease progression, real world data (RWD), rare disease

## Abstract

The 21^st^ Century Cures Act requires FDA to expand its use of real-world evidence (RWE) to support approval of previously approved drugs for new disease indications and post-marketing study requirements. To address this need in neonates, the FDA and the Critical Path Institute (C-Path) established the International Neonatal Consortium (INC) to advance regulatory science and expedite neonatal drug development. FDA recently provided funding for INC to generate RWE to support regulatory decision making in neonatal drug development. One study is focused on developing a validated definition of bronchopulmonary dysplasia (BPD) in neonates. BPD is difficult to diagnose with diverse disease trajectories and few viable treatment options. Despite intense research efforts, limited understanding of the underlying disease pathobiology and disease projection continues in the context of a computable phenotype. It will be important to determine if: 1) a large, multisource aggregation of real-world data (RWD) will allow identification of validated risk factors and surrogate endpoints for BPD, and 2) the inclusion of these simulations will identify risk factors and surrogate endpoints for studies to prevent or treat BPD and its related long-term complications. The overall goal is to develop qualified, fit-for-purpose disease progression models which facilitate credible trial simulations while quantitatively capturing mechanistic relationships relevant for disease progression and the development of future treatments. The extent to which neonatal RWD can inform these models is unknown and its appropriateness cannot be guaranteed. A component of this approach is the critical evaluation of the various RWD sources for context-of use (COU)-driven models. The present manuscript defines a landscape of the data including targeted literature searches and solicitation of neonatal RWD sources from international stakeholders; analysis plans to develop a family of models of BPD in neonates, leveraging previous clinical trial experience and real-world patient data is also described.

## Introduction

Bronchopulmonary dysplasia (BPD) is a chronic inflammatory lung disease that affects thousands of neonates and infants every year (Steinhorn et al., 2021). The pathophysiology and severity are characterized by the need for supplemental oxygenation or ventilatory support at 36 or 40 weeks post-menstrual age (PMA). BPD represents disruption of normal lung development before the saccular stage (before 32 weeks PMA), corresponding to a crucial time in the formation and architectural development of alveoli. Risk factors are numerous including: internal factors (prematurity, gender, genetics, *in utero* tobacco exposure/growth), iatrogenic factors (mechanical ventilation or/and oxygen supplementation, blood transfusions), or external factors (antenatal or/and postnatal infection, intra-uterine growth restriction). Although it is largely accepted that BPD results from lung damage and inflammation/oxidation triggered by mechanical ventilation and hyperoxia, the specific molecular mechanisms that result in compromised lung function and arrested development remain unknown. BPD likely represents several heterogeneous endotypes, with multi-hit processes likely (Niedermaier and Hilgendorff, 2015; Thébaud et al., 2019). Chest radiographs, blood tests, and echocardiograms (to assess the presence of pulmonary hypertension) may also be helpful to evaluate prognosis but can be non-specific. Preferred endpoints have varied significantly over decades (Cuevas Guaman et al., 2021) and there has been very little work on intermediate timepoints needed to recognize disease progression. While recent advances in neonatal care have improved the survival of very low-birthweight infants, the rates of BPD have not improved accordingly. This is mainly due to our limited understanding of the pathogenesis and the lack of effective therapeutic options currently available.

The definition of BPD has been constantly evolving over the past 30 years, with up to 18 separate definitions reported in the literature (Steinhorn et al., 2021). Some of the evolution in the definition of BPD is tied to changes in etiology that have resulted from advances in neonatal care, such as antenatal corticosteroid administration and postnatal surfactant therapy, which have improved the survival of extremely premature infants. What has been described as “old” BPD, often linked to lung damage and fibrosis from injury induced by oxygen toxicity and barotrauma from prolonged mechanical ventilation, has become less common than the concept of “new” BPD focused more on grow arrest and disordered lung development. Much of the discussion also involves establishing a definition that correlates with pulmonary outcome later in infancy and childhood. Of course, all definitions need to be put in the context of the current standard of care and treatment strategies which have changed considerably over time.

In response to a recent grant award from FDA, the International Neonatal Consortium (INC) has an opportunity to collate and evaluate real world data (RWD) sources to assess their value in informing various aspects of neonatal drug development. There is a greater recognition that such data may provide an important key to assess the heterogeneity of neonatal populations as well as the significant variability in respect to the current standard of care (Horton et al., 2021). While this report provides a broad landscape of the available data sources to inform a family of models to assess BPD disease progression, it is the RWD that represents the new Frontier in this effort. A key deliverable from the INC RWD grant (1 U01 FD007220-01) is the development of a universal definition of BPD that will serve as the anchor and baseline for models that capture the relevant disease biology and quantify disease progression over time.

**FIGURE 1:**
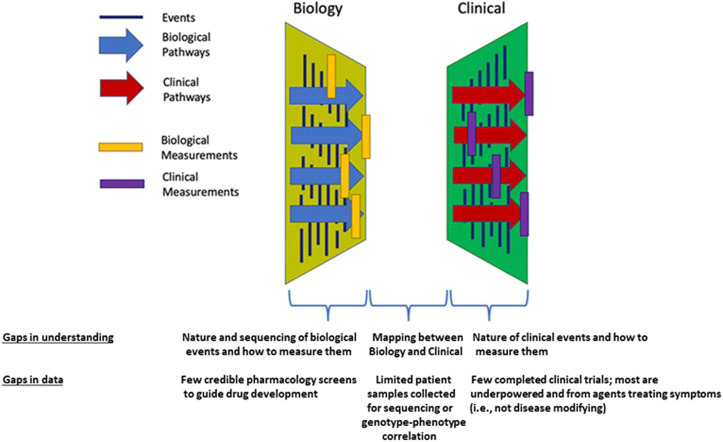
Conceptual framework for the necessary connections and current knowledge gaps between biologic and clinical data which exist for BPD.

**FIGURE 2:**
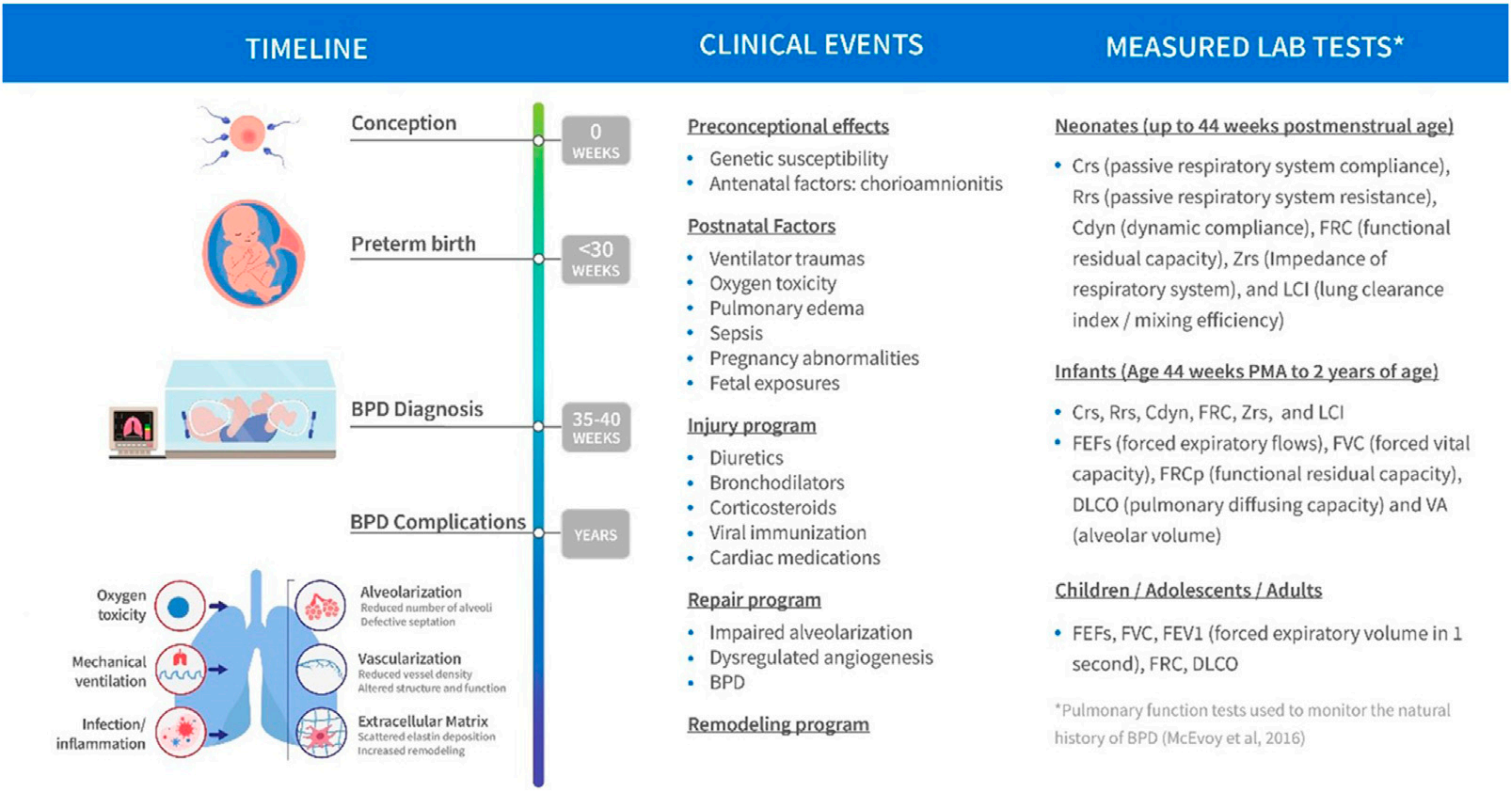
Schematic of BPD disease progression with variables of clinical interest linked to stage of progression.

The current state of clinical and biological resources that would facilitate a bridge to better understand BPD is conceptualized in Figure 1. Biological events and processes underpin the short- and long-term clinical manifestations of BPD. Techniques to measure biological events and mechanisms have not been delineated or deployed at sufficient scale to provide a comprehensive “map” of the condition. Similarly, clinical events have not been defined other than a variety of short- and long-term endpoints. Clinical observations are not informed by the timing or nature of biological processes or mechanisms. In other conditions, information about the stages of pathophysiology (biological processes) and clinical events inform the development of therapeutic options. Data from biological and clinical sources, summarized in Figure 2, can be combined in “disease progression models” (DPM) that capture the stages of disease development, the timing of the stages, and the extent of variation between individuals in the pathway to disease. DPM are a key tool in drug development allowing rational targeting of interventions and evidence-based planning of clinical trials (Fouarge et al., 2021; Barrett et al., 2022). Here we review the DPM concept applied to strategies for the development of a BPD DPM. This manuscript seeks to both prospectively assess the potential of the clinical real-world data to inform BPD (and therefore, other complications of extreme prematurity) definition and also the potential of utilizing such data to construct models that would inform BPD drug development as a context of use.

## Methods—literature review

Underpinning this landscape data analysis is a narrative review of BPD DPM. The search for 1990–2021 peer-reviewed articles via the National Library of Medicine’s PubMed site included Academic OneFile, JSTOR, Sage Journals, and related databases [including Scopus and the Directory of Open Access Journals (DOAJ)]. Google Scholar was also utilized to locate open access articles. MeSH terms included the following: Animals; Animals, Newborn; Bronchopulmonary Dysplasia/metabolism*; Bronchopulmonary Dysplasia/pathology*; Disease Models, Animal*; Humans; Infant, Newborn; Infant, Premature/growth and development; Infant, Premature/metabolism*; Lung/growth and development Lung/metabolism*; Lung/pathology*; Rabbits. Selected references identified by this search were supplemented by papers from the authors’ collections and identification of additional resources among subject matter experts from the INC. The INC BPD working group and modeling and simulation sub-team filtered the literature search results into categories that would include one of the following: relevant data from which model priors could be abstracted, published models of various types (e.g., predictive, descriptive, mechanistic, etc.), descriptive and/or quantitative definitions of BPD to be used as comparators for a future definition.

## Baseline animal model evaluation

There have been numerous efforts to develop appropriate animal models of BPD to improve our understanding of the disease origin, progression, treatment, and prevention. These animal models have been explored in a broad range of species (Salaets et al., 2017; van der Merwe et al., 2021). Mice and rats are commonly used for models of BPD (e.g., chronic hyperoxia) due to ready availability, low cost, short gestational durations, and large litter sizes. Full-term mouse and rat pups are born during the saccular stage, which aligns with lung development in preterm neonates. However, despite term mouse and rat lungs being structurally underdeveloped, they are functionally mature and do not require respiratory support similar to preterm neonates at risk for the development of BPD, thereby limiting the translatability of these models. A well-constructed quantitative systems pharmacology (QSP) model often relies on animal models to help define relationships with key moieties of interest. This contributes to disease progression or is involved in the cascade of events that define a target molecule’s interaction with physiologic organs, tissues, and cells of interest. Rate constants involved in the various reaction kinetics are often scalable across species and can be approximated in humans with appropriate allometric scaling techniques.

Several mouse and rat studies have contributed to much of the current understanding of the pathogenesis of BPD and identified important signaling molecules that can be used to inform semi-mechanistic mathematical models of disease progression (Tawhai and Burrowes, 2003; Zhou et al., 2019). These signaling molecules are relevant biomarkers that are used as indicators of inflammatory changes and immune events and include neutrophils, monocytes, inflammatory cytokines/chemokines, matrix proteins, growth factors, etc. These biomarkers can be found in various biofluids such as blood, urine, and bronchoalveolar lavage.

Following hyperoxic exposure, many species develop inflammation and alveolar simplification like that observed in rats and mice. Increased concentrations of pro-inflammatory cytokines and chemokines are likely central mediators of the response to many noxious stimuli. These studies can be used to calibrate mathematical models of the pro- and anti-inflammatory response dynamics and support the translation of these models across species. The rodent experiments have also been useful for the design of large animal trials including non-human primates.

Non-human primates are the most translationally relevant models of BPD, but account for the fewest studies available due to steep costs and ethical concerns (Nardiello et al., 2017). Rabbits, pigs, and sheep fill the gap between rodents and primates (D'Angio et al., 2016). The baboon experience has been beneficial for studying short-term BPD disease progression including providing clinically credible lung function and histology data (Coalson et al., 1982; Yoder and Coalson, 2014). The preterm baboon model does permit investigation of molecular pathways and genetic regulation of inflammatory processes in the developing lung (Yoder and Coalson, 2014) though there is likely less interest in keeping these colonies viable recently given the cost of maintaining.

Despite the number of animal models, no clinically relevant and standardized model exists, leaving several gaps that must be addressed to optimize the pathophysiology and treatment of BPD (Wickramasinghe et al., 2021a). The vast majority of preclinical models of BPD aim to achieve a simplified alveolar structure with disordered surrounding vasculature in order to study relevant mechanisms of malformation. A consequence of focusing on disease onset is that these preclinical models have yet to expand into longitudinal frameworks that connect to long-term outcomes. Few studies continue experimental observation beyond the well-controlled period of pathogenic insults due to the extreme financial and time commitments (Yoder and Coalson, 2014; Wickramasinghe et al., 2021a). The introduction of an intermittent insult methodology that combines injury and repair phases to align with clinical protocols more closely has expanded some study durations, but not enough to capture long-term sequelae of the disease (Ratner et al., 2009). Additionally, there is a lack of standardized approaches to introducing noxious stimuli across the range of investigated species, which highlights the broad spectrum of insults that have been explored but simultaneously restricts translational comparability. Establishing standardized protocols could facilitate a more systematic review of the direct impact that various insults have on lung development. While many studies report structural and histological findings that demonstrate alveolar simplification, few are accompanied by physiological metrics of gas exchange (Reynolds et al., 2010; Wickramasinghe et al., 2021a). Including any functional metrics would add a quantitative layer to these studies and help elucidate structure-function relationships throughout lung development, which would be useful from a longitudinal mathematical modeling standpoint. Lastly, although pulmonary dysfunction is the primary outcome explored in many animal models of BPD, the downstream complications involving other organ systems is not well-defined. Neurodevelopmental impairment and retinopathy are known comorbidities likely linked to a dysregulated immune response and should be considered along with changes in other organ systems in future animal models of BPD (Wickramasinghe et al., 2021b). An examination of the extrapulmonary organs in these animals will facilitate a more complete picture and facilitate an improved understanding of BPD pathophysiology and progression.

Further complicating the feasibility of developing a QSP-type model of disease progression in BPD is the classification of BPD as a syndrome rather than a disease. Without clearly defined endotypes and phenotypes, establishing a link between the two using mechanistic and quantitative terms is not possible. The complex mechanistic interactions induced by the various noxious stimuli contribute to the heterogeneity of disease trajectories and complicate the classification into clinical subtypes or phenotypes (Wu et al., 2020). The fields of genomics and metabolomics hold promise for identifying unique signatures of specific interactions or patterns that could be used for classification. Two recent studies have explored the complex intracellular dynamics that occur during the transition to air breathing by using single-cell RNA-sequencing (scRNA-seq) to generate cellular composition maps and identify biologically plausible pathological pathways (Hurskainen et al., 2021; Zepp et al., 2021).

Several of the animal models aim to identify the basic mechanisms of late lung development by inducing alveolar simplification and vascular irregularities, hallmarks of the new BPD. These studies have uncovered a multitude of new mechanisms of normal and dysregulated lung development. One recent example in the area of lung cellular and molecular physiology is a study that probed the role of oxygen and steroids (e.g., dexamethasone) in the regulation of surfactant secretion by alveolar epithelial type II cells (AEC2s). Htun et al. (Htun et al., 2021) proposed a mechanism in support of the observed effect of glucocorticoids in increasing surfactant secretion through suppression of components within the natriuretic peptide system of AEC2s. Still, the interplay of these molecular and cellular pathways involved in lung development, injury, and repair remains complex and the influence on arresting proper lung maturation is not fully understood.

To date, the existing preclinical model data has not been collectively assembled in a meaningful way. This is an essential step for the initiation of a multi-scale QSP model that would provide a more clinically structured disease progression model for BPD. This initial landscaping effort has incorporated a preclinical data coordination plan to support the family of models that will support efforts to evaluate RWD sources that support regulatory decision making.

## Results

### Current development of therapeutic options to treat BPD

Current therapeutics for BPD and RDS involve ventilatory management, steroids, and administration of various agents such as pulmonary surfactant, caffeine, vitamin A, nitric oxide, diuretics, and stem cells (Michael et al., 2018). Some are only at early stages of evaluation and only steroids, vitamin A and caffeine are the only interventions that have shown to reduce BPD based on RCTs. The efficacy of these agents in preventing and ameliorating BPD varies depending on the populations studied and the timing of the intervention(s). Some of these agents have been developed opportunistically rather than through a planned application of insights from preclinical work. Since there are few pharmaceutical sponsors who would conduct and store such data, the typical preclinical safety, pharmacology, and PK/PD data for these agents is sparse or nonexistent. Published preclinical investigations suggests multiple therapeutic targets are relevant (Bhandari, 2014). Planned application of preclinical work is also hampered by the lack of an accurate quantitative description of the current and evolving standard of care, which can be defined and codified in a DPM. This could be the starting point for an RWD-informed clinically focused standard of care baseline model from which drug treatment models could be compared. A non-mechanistic trajectory of disease progression from time of diagnosis could be constructed to complement future clinical trial simulations.

### BPD disease progression

It is unclear when BPD begins, but many believe its origins occur *in-utero* (Taglauer et al., 2018). It would seem to depend on perinatal history as severe documented chorio or preeclampsia with associated IUGR do initiate BPD before birth. Detailed assessment of neonatal pulmonary function after a preterm delivery is difficult but would offer great value to understand the evolution of disease and identify potential windows of vulnerability and intervention. In preterm neonates, lung development that would normally occur *in-utero* happens postnatally under altered mechanical and environmental conditions. This includes active tidal breathing with strain/stretch of immature intrathoracic structures and a state of relative hyperoxia (even in room air). Lung development is also affected by conditions precipitating preterm delivery, including inflammation and infections. While preterm delivery impacts normal alveolarization and pulmonary vascularization, it can also affect mechanical processes in the lung (McEvoy and Aschner, 2015). As they mature, individuals manifest with ongoing respiratory symptoms and reduced lung function, with pulmonary function tests (PFTs) showing expiratory flow limitation at school age (which may respond to bronchodilators) and into adulthood. There is concern that BPD will predispose to chronic obstructive pulmonary disease (COPD) since infants are beginning life with reduced lung function and longitudinal cohorts indicate that individuals track along their predetermined PFT percentiles throughout life.

Due to advances in neonatal care, increased numbers of preterm neonates are surviving at lower gestational ages. Up to half of extremely low birth weight infants may develop BPD. Owing to paucity of evidence and absence of comprehensive guidelines for outpatient management, there is significant variation in management. Additionally, the only validated phenotypes for preterm respiratory disease are at a single timepoint (36 weeks corrected gestational age). Work is needed to define the respiratory outcomes for individuals born preterm over their lifetime.

### Models for BPD

Quantitative models fall in many categories and offer value to drug development in a variety of ways. In the context of a model-informed drug development (MIDD) approach, models can be developed to de-risk decision making at various stages of drug development. These include quantitative system pharmacology (QSP), pharmacokinetic (PK), pharmacokinetic-pharmacodynamic (PK/PD), physiologically based pharmacokinetic (PBPK), pharmacometric (PMX), clinical trial simulation (CTS) and pharmacoeconomic models. Several of these foundational models may be incorporated into a clinical trial simulation paradigm used to project the probability of technical success (PTOS) for a proposed trial design. A range of models have already been developed and published to support BPD research and clinical disease management (Table 1).

**TABLE 1:** Previously published BPD prognostic and quantitative models to be utilized for qualification of future BPD disease progression model.

Model (Reference)	Category	Data Sources	Purpose
Review of 23 clinical prediction models (Onland et al., 2013)	Regression-based models; multivariate analyses; retrospective and prospective	A variety of data sources including the PreVILIG database	• Review the quality and validity of models that predict BPD in preterm infants using clinical information from the first week of life
			• Attempted to externally validate models and compare predictors identified
BPD severity prediction model (Valenzuela-Stutman et al., 2019)	Binary logistic regression models to evaluate the predictive value of different variables, using respiratory hospitalization as the primary outcome	• Primary cohort included 188 premature infants (≤32 weeks PMA) admitted to the NICU at Children’s National Health System (CNHS) in Washington, D.C.	• Approach improved BPD risk assessment, particularly in extremely premature infants.
		• The validation cohort included 130 premature infants (≤36 weeks PMA) admitted to the NICU at The Hospital Militar Central and the Hospital Universitario Clinica San Rafael in Bogota, Colombia.	• Internal validation included lung X-ray imaging and phenotypical characterization of BPD severity levels.
			• External validation conducted in an independent longitudinal cohort of premature infants (≤36 weeks PMA, n = 130; Bogota).
BPD risk prediction model (Alvarez-Fuente et al., 2019)	Multivariate logistic regression model to identify risk factors for BPD development by determining the odds ratio of both groups, no-BPD versus BPD, in relation to clinical, echocardiographic and analytic factors	• 5 Spanish hospitals: 50 patients with a median gestational age of 26 weeks and weight of 871 g (range 590-1200g).	• Study and model aimed to explore the ability of clinical, echocardiographic and analytical variables to predict moderate or severe BPD in a cohort of extremely preterm infants.
BPD severity prediction (Taglauer et al., 2018)	Forward logistic regression models with predictive values evaluated using a ROC curve	Multicenter study including 16,407 infants weighing 500-1500 g (2001-2015) from the Neocosur Network	• Predictive power models for moderate/severe BPD and BPD/death at four postnatal ages.
			• Birth weight contributed the most in explaining BPD, followed by GA and 1-min Apgar score
BPD Risk factors in preterm infants (Alvarez-Fuente et al., 2019)	Multiple logistic regression analysis: sensitivity and specificity of the model assessed by ROC curve	Seventy-two preterm infants (30 with BPD and 42 non-BPD controls) admitted in the NICU of the Children's Hospital of Soochow University during 2017 enrolled; prospective longitudinal study	• To identify postnatal risk factors for bronchopulmonary dysplasia (BPD) development in preterm infants with gestational age ≤32 weeks
			• Perinatal data, a neonatal critical illness score (NCIS), different soluble B7-H3(sB7-H3), and interleukin-18 (IL-18) levels by days after birth collected; early predictive model for BPD development established
Mechanistic model of gas exchange and ventilation under a broad range of local and systemic inflammatory stimuli (Reynolds et al., 2010)	Diffusion of oxygen and carbon dioxide, hemoglobin uptake of oxygen, and enzymatic reactions governing carbon dioxide and bicarbonate levels.	ODE-based multi-scale model based on literature priors	• Simulation model of pulmonary function under inflammatory stress and of interventions aimed at improving gas exchange in this broadly relevant context.
			• Generically multiscale model to be improved in its physiologic accuracy and computational load.
Integrative anatomically-based model - incorporates descriptions of material properties and anatomical structure at a range of levels of interest (Tawhai and Burrowes, 2003)	Finite element meshes of the lung lobes, airways, blood vessels, parenchyma, and microcirculation.	Database of publications, models, and data related to the pulmonary circulation	• Provides a framework for quantitative description of a system’s geometry, behavior, or interactions. Laboratory observations are incorporated into the models as, for example, geometric data or rate constants, and experimentation is also used to validate the model’s performance.
			• “Lung Atlas” is currently being developed based on structural and functional computed tomography (CT) imaging (Alvarez-Fuente et al., 2019)

Several predictive models are available for BPD. Most predictive models include several BPD risk factors, such as birth weight, GA, chorioamnionitis, preeclampsia, respiratory parameters, etc. (Ding et al., 2020; Valenzuela-Stutman et al., 2019; Nino et al., 2020). These known risk factors increase neonatologists’ awareness of the potential risk of BPD in selected patients but are still not able to universally identify patients with a high risk of developing moderate to severe BPD and tend to overestimate this risk. This makes it difficult to implement early interventions for selected patients who will, with high probability, develop the most severe disease.

### Disease progression models

In general, modeling progression of chronic diseases enables better understanding of disease prognosis and provides insights into staging systems. This approach could assist early diagnosis and personalized care and facilitate the development and evaluation of interventions. Other types of models, including disease progression and quantitative systems pharmacology models, have the potential to provide more mechanistic understanding of disease biology in the context of development, maturation, and other time dependencies. This assumes that the data supporting these relationships is of sufficient quantity, diversity, and quality. To date, there are few quantitative models other than the predictive models shown in Table 1 and no disease progression models for BPD.

To characterize the natural progression of disease, these models generally incorporate longitudinal data for biomarker(s) of disease severity or can incorporate more direct measures of disease severity. Although such data are unlikely to be collected during routine clinical care, there is some hope that laboratory measures currently monitored in BPD may be suitable for that purpose. Disease models are also often linked to PK–PD models so the influence of drug treatment on disease progression can be quantified and evaluated. Once again, it is important to note that there are no well-established and effective agents for the prevention and/or treatment of BPD reflected in the current standard of care. However, there may be an opportunity to optimize dosing of current treatment options that shift or mitigate BPD progression.

Semi-mechanistic models are a particular class of disease progression models with great potential to impact BPD. These models are both data-driven (e.g., fully empirical models) and grounded in biological and pathophysiological processes similar to traditional systems models. To effectively combine both approaches and to achieve the optimum balance between parsimony and goodness-of-fit, the model is limited to the most critical processes that are necessary to explain the relevant data. In the case of a well-developed model of BPD disease progression, the key processes to consider are the complex inflammatory pathways that result from both genetic and environmental triggers and the processes involved with the structural and functional changes that occur in the lungs of the preterm neonate. In BPD, oxidation and inflammation are the common denominators that link genetic and environmental factors associated with disease severity. From this etiological perspective, a semi-mechanistic model could provide an avenue to interrogate the interplay of infection, hyperoxia, and barotrauma/volutrauma with the structural and functional changes observed in the lungs of individuals with BPD. The stages of lung development are well-delineated, but the relationship between the development of the immune response and resolution at the various stages of lung development is not fully understood (Kolls, 2017). From a drug development context much of this knowledge is focused on adult lung disease and related conditions where financial incentives are easier to define which is also a motivation herein to provide a context and framework from which BPD can be better defined and acted upon.

Relevant biomarkers can be used to calibrate a model that incorporates inflammatory pathways and to probe for the presence of links between the pro- and anti-inflammatory imbalance and the emergent phenotype of alveolar simplification and dysregulated vascularization observed in BPD (Bhandari, 2014; Balany and Bhandari, 2015). Some inflammation-related biomarkers that could support this future study include cytokines/chemokines, reactive oxygen and nitrogen species, as well as growth factors and other mediators. It is possible that disease endotypes exist that are defined by different drivers of inflammation and different response profiles, but still give way to the same disease phenotype. If true, modeling could be used to classify the endotypes and to subsequently explore potential therapeutic regimens unique to the identified endotypes. It may also be possible to link such a model to clinical outcomes like PFTs or a more discrete outcome like the probability of developing moderate to severe BPD. Yet, developing another predictive model without a mechanistic link to BPD disease progression is not enough.

**FIGURE 3:**
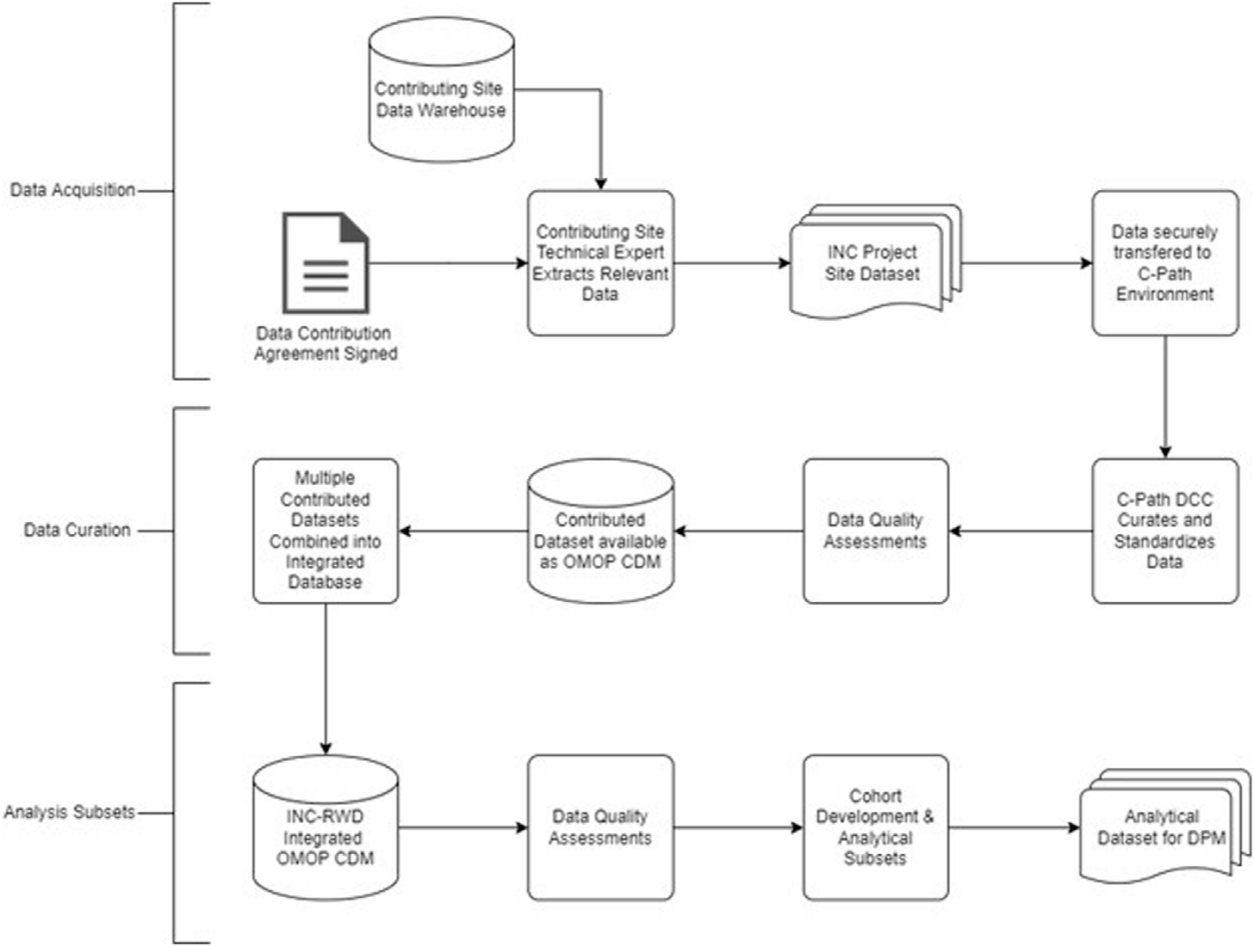
Data flow diagram to generate analytical subset from contributed real world data.

The INC RWD project will develop a variety of models that facilitate a quantitative description of BPD. This reflects the underlying pathophysiology and disease biology, not only in the context of the available data, but also future data types that could be collected during routine clinical care as well as in the conduct of future clinical trials. Well-curated RWD from neonates can contribute to the validation of quantitative models of symptom progression and facilitate the development of useful insights and the generation of RWE. The workplan is summarized in Table 2. Figure 3 includes a data flow diagram describing the process from data acquisition through development of a dataset used for model development.

**TABLE 2:** Proposed workplan—critical requirements of the BPD disease progression model effort based on initial scoping from INC working group.

Task	Details
Modeling strategy plan	• Identify family of models that would facilitate clinical decision making and the design of clinical trials in neonatal BPD patients (likely to include QSP, disease progression, clinical utility, and clinical trial simulation models)
Develop quantitative clinical attributes that can be used to define BPD patient phenotypes	• Using a “reverse engineering” approach applied to published clinical prediction models (see Table 3), identify clinical variables form various RWD sources and values / ranges identified with BPD severity. Explore boundaries and patient selection via sensitivity analysis.
Identify data sources and elements that would be utilized in each target model type	• From the varied RWD sources (See Table 3), identify presence of data used to develop and validate the various model types.
Verify data quality concerns based on “fit-for-purpose” approach	• Assess data quality metrics for each of the identified sources likely to contribute to uncertainty in the model development and the fidelity of the final model and model predictions.
Conduct simulations to verify model operating characteristics	• Explore boundary conditions of key variables, model parameters and response predictions.

### Data requirements

#### Outcomes

Well-defined outcomes are essential for the development of DPM. DPMs need to progress towards an outcome to select and weigh variables included in the model. The definitions of clinical endpoints have been “definitions of convenience” that are used to inform clinical practice, benchmarking, and epidemiology. Extant endpoints are based on time points and assessments that have clinical validity. These clinical definitions have not been validated for reproducibility or prediction of long-term outcomes and do not reflect biological mechanisms or key events in disease progression. The lack of uniform definitions for clinical endpoints has prevented effective meta-analyses among existing therapeutic studies of BPD (Cole et al., 2010). In addition, the lack of a consistent, well-founded definition also hinders the development of new therapies. From a modeling perspective, one consideration is that clinical SMEs can be used to define credible patient profiles from a combination of existing RWD observations and plausible/credible models so these model-based virtual patients can be used to qualify components of the model as its being assembled.

#### Data sources: RWD and clinical trials

In one sense neonatology is a data-rich specialty since neonates are monitored closely in the NICU. This fact informs the belief that RWD can inform the development of DPM and important outcomes despite the lack of adequately powered, neonatal clinical trials. The availability of RWD reflecting the diversity in populations and disease stage can also contribute to the generalizability of the model. To date, neonatal RWD has not been collated from multiple sources.

Randomized clinical trial data can be used to drive the development of empirical models of BPD disease progression that rely more heavily on statistical methods rather than the mechanistic underpinnings of systems-based approaches. Data can be pooled across multiple studies and population-based methods can be utilized to explain the observed interindividual subject variability on the baseline severity and rate of disease progression. These methods incorporate covariates and patient characteristics from the available data to describe the variability and to identify sub-types of the disease that may respond differentially to a given treatment. Mathematical models that account for the diversity in a disease population can be used to generate powerful clinical trial simulation tools for trial design optimization (Barrett et al., 2022).

Unfortunately, the portfolio of new drugs or other modalities to treat BPD is small and legacy trials with historical agents have been largely underpowered and have focused on symptomatic relief. This is primarily due to the lack of phenotypic discrimination and uncertain disease progression. A variety of agents have been developed or re-purposed to target different points in the pathways that lead to BPD, including anti-inflammatories, diuretics, steroids, pulmonary vasodilators, antioxidants, and molecules involved in the cell signaling cascade thought to be involved in the pathogenesis of BPD. IL1RA, glyburide, and inhaled budesonide are currently the most promising anti-inflammatory therapies that have the potential to prevent BPD in preterm infants. However, more studies will have to investigate the safety and potential long-term effects in human neonates. Another aspirational emphasis of this work is to leverage the relevant knowledge from adult lung disease and drug development tools to facilitate the BPD data, model and drug development tools landscape so that an easier and perhaps less costly roadmap to development can be defined.

#### Data collection

Data will be collected from clinical trials and real-world data sources including Electronic Health Records, clinical registries, observational studies (Table 3). Contributing organizations include members of the International Neonatal Consortium and I-ACT. Working in partnership with C-Path’s Data Collaboration Center (DCC), the contributing organizations will develop and execute queries to extract data from the EHR, clinical data warehouse, or other research databases and registries as appropriate. Data elements that will be included in the data extracted were identified by subject matter experts as being relevant to the clinical presentation of BPD. For data from electronic health records, contributing organizations have been asked to develop cohorts that included records from neonates and their mothers who were admitted to the neonatal intensive care unit between 22- and 42-week gestational age. As a component of the data extraction process contributing organizations have been asked to remove identifiable information from their datasets and apply de-identification methods to the source data to protect the privacy of the patients. Data will be securely transferred to the DCC where an integrated database will be assembled, and data validation will occur. The DCC will store the data in secure environments with appropriate access-based controls to minimize the risk of data breach and conduct additional assessment of the data to ensure identifiable data elements have been removed to protect patient privacy.

**TABLE 3:** RWD available to construct BPD Disease Progression Model from committed sources to the recent INC / C-Path Grant with FDA.

Contribution Organization—Dataset Type
*Dataset Name*
Tufts Medical Center—Electronic Health Records
Nagano Children's Hospital—Electronic Health Records
Kyorin University—Electronic Health Records
Driscoll Children's Hospital—Electronic Health Records
Prisma Health Children's Hospital—Electronic Health Records
Children's Hospital of Philadelphia—Electronic Health Records
University of New Mexico Health Sciences Center—Electronic Health Records
University of Texas Health San Antonio—Electronic Health Records
Anne & Robert H. Lurie Children's Hospital of Chicago—Electronic Health Records
Prentice Women's Hospital/Northwestern—Electronic Health Records
University of Utah Health Science Center—Electronic Health Records
UPMC Children's Hospital of Pittsburgh—Electronic Health Records
Mount Sinai Hospital (Canada)—Electronic Health Records
University of Minnesota: Masonic Children's Hospital—Electronic Health Records
Children's Mercy Hospital Kansas City—Electronic Health Records
Tufts Medical Center—Clinical Trial
*Efficacy of Recombinant Human Clara Cell 10 Protein (rhCC10) Administered to Premature Neonates with Respiratory Distress Syndrome*
Tufts Medical Center—Observational Study
*Improving Bronchopulmonary Dysplasia (BPD) Predictors and Outcomes for Clinical Trials (STOP-BPD)*
NICHD DASH / NRN—Clinical Trial
*Surfactant Positive Airway Pressure and Pulse Oximetry Trial (SUPPORT)*
Children's Hospital Colorado—Observational Study
*Genetic Basis for Impaired Angiogenic Signaling in BPD*
Chiesi Pharmaceuticals—Clinical Trial
*A Study to Investigate the Safety, Tolerability and Efficacy Of Nebulized Curosurf In Preterm Neonates With Respiratory Distress Syndrome (RDS)*
Chiesi Pharmaceuticals—Clinical Trial
*A Double Blind, Randomized, Controlled Study to Evaluate CHF 5633 (Synthetic Surfactant) and Poractant Alfa in Neonates with Respiratory Distress Syndrome (RDS)*
Chiesi Pharmaceuticals—Clinical Trial
*European Non-Interventional Post-Authorization Study to Assess Drug Utilization and Safety Of Caffeine Citrate (Peyona) In Treatment Of Premature Infants*
NICHD DASH / NRN—Clinical Trial
*Inhaled Nitric Oxide for Preterm Infants with Severe Respiratory Failure (Preemie iNO)*

#### Data integration

Given the breadth and variation of source data structure and data element representation, there are technical challenges with developing a DPM due to the lack of interoperability of source data. In order to ease these challenges, we will standardize the structure, content, and semantics of the data to make it possible to modeling of all data sources with a uniform approach. The INC project will use the Observational Health Data Sciences and Informatics (OHDSI) program’s Observation Medical Outcomes Partnership (OMOP) Common Data Model (CDM) (Stang et al., 2010) as the data model used to integrate datasets for this project. The OMOP CDM provides a consistent and reliable data model to represent all observational data and has an extensive set of vocabulary mappings to a hierarchical vocabulary of concept sets. In cases where a contributing organization has an existing mapping to a common data model, these will be requested and the DCC will work with local experts to confirm these mappings contain the variables identified by the working group. In the scenario that a site does not have a mapping to a CDM the DCC will request the data in the current format and then conduct all data transformation activities locally. After validating transformation of the data to a CDM the data will be loaded into a common database. Data validation will occur on both the source data and the data transformations.

#### Data quality assessment

In addition to the technical challenges surrounding the integration and interoperability, data generated from real-world settings are intended for operational use and not optimized for research. The lack of systematic data collection, errors introduced by system bugs or human mistakes, and ambiguous data definitions raise concerns about the utility and reliability of the real-world data. A critical piece of a generating evidence from RWD will assessing the quality of the data and ensuring “fitness for use” within the context of the disease.

The INC project will leverage previous work and lessons learned from data quality frameworks available in the literature (Kahn et al., 2012; Kahn et al., 2016; Khare et al., 2017; Khare et al., 2019; Liaw et al., 2021) and existing software packages to guide our assessment. Standardizing the data into the OMOP CDM has an additional benefit of a data model that lends itself to assessing data quality and the availability of robust open-source projects supported by the community to evaluate data quality such as the Data Quality Dashboard which includes over 3,300 data quality checks (Blacketer et al., 2021; Liaw et al., 2021). The assessment will focus on verifying and validating the conformance, completeness, and plausibility of the data. Using these assessments will help identify erroneous records and provide an overall assessment of the reliability of both each dataset prior to combining multiple datasets into a single integrated data model and after the integration has occurred. The assessments will include data quality checks that cover a range of factors that could contribute to the overall “fitness for use”. Because the multitude of sources of errors, the assessments will evaluate multiple dimensions of the data from multiple perspectives. One key feature is ensuring the integrity of data types. For example, evaluating that the numeric data fields include only numeric values and that the values themselves are plausible given biological or temporal restraints on the range of values that could exist. A related data quality check will need to assess the range of values given the measurement unit for a given observation. Additional data checks will test the temporal reliability and plausibility of the data and the related records within the data. For example, these checks will evaluate if records reporting the use of respiratory support devices have dates that occur after the date of birth.

Another important aspect of these assessments includes evaluating the completeness of data both in the level of value missingness but also in the coverage of key data elements. For example, ensuring that the data provided by a contributing institution includes data elements which are relevant to developing a disease progression model and accurately describing the patient’s engagement with the health care facility. In cases where the completeness of data is lacking it will be important to contextually understand if these data are missing due to errors in the extraction process or the lack of availability in source systems and to incorporate appropriate statistical error estimation in the disease progression model.

One unique challenge of the INC project is the integration of multisite data. The process of combining multiple data sources poses unique challenges especially from a data quality perspective. It is possible for each individual sites dataset to pass the data quality assessments but when compared to datasets from other sites there are discrepancies that result in it not being reasonable to combine the dataset. These discrepancies may occur due to the characteristics and statistical distributions of the data being significantly different and explainable by understanding local site clinical practices or they may be due to semantic irregularities in the data. In the case of variation introduced by site specific protocols these instances will need to be reviewed to determine if it is appropriate to still combine the data. In instances of semantic irregularities these may be able to be resolved by further data curation of the data elements. Previous work has described the implications of research networks and combining multisite datasets that will guide the development of key assessments (Kahn et al., 2012). To assess variations in data characteristics across multisite datasets we will calculate and evaluate comparative descriptive statistics for data elements that are important for the fitness of use to identify anomalies in data patterns.

#### Analytical dataset and cohort subsets

After combining the source datasets into an integrated database an analytical subset will be generated for use in generating the disease progression model. The development of this analytical subset requires identifying similar patient cohorts for comparison and selecting covariates that are appropriate for the model. Identifying patient cohorts will rely on defining computable phenotypes that can be applied to the patient population. A computable phenotype includes clinical characteristics defined by a set of data elements and logical expressions that can be understood by a machine and electronically queried to identify similar patients within a population (Richesson et al., 2013). In addition to phenotypical relationships, details such as observation periods, time-at-risk, completeness of data, and density of data will play important roles in identifying a subset of patients into a cohort. Groups of patients will be identified to comprise of comparator and outcome cohorts that will compromise the patients and observations to be used during data analysis.

#### Initial modeling efforts

A multidisciplinary team comprised of clinical subject matter experts including clinical care givers, clinical trialists, researchers, and quantitative scientists including experienced modelers, data scientists and engineers have participated in monthly meetings to develop the workplan, assemble and advise on the relevant datasets and assess the data availability, suitability, and quality for the proposed DPM context of use. With the focus of constructing a BPD QSP model that can represent a mechanistic anchor from which a future BPD DPM can be assembled, a multiscale approach was proposed with the following goals: 1) describe the relevant physiologic landscape involved with BPD disease progression (e.g., lung, GI tract, and immune system), 2) define states/conditions which define the “healthy” versus “disease” states, and 3) describe maturation and developmental considerations which include different patient phenotypes and disease roadmaps.

**FIGURE 4:**
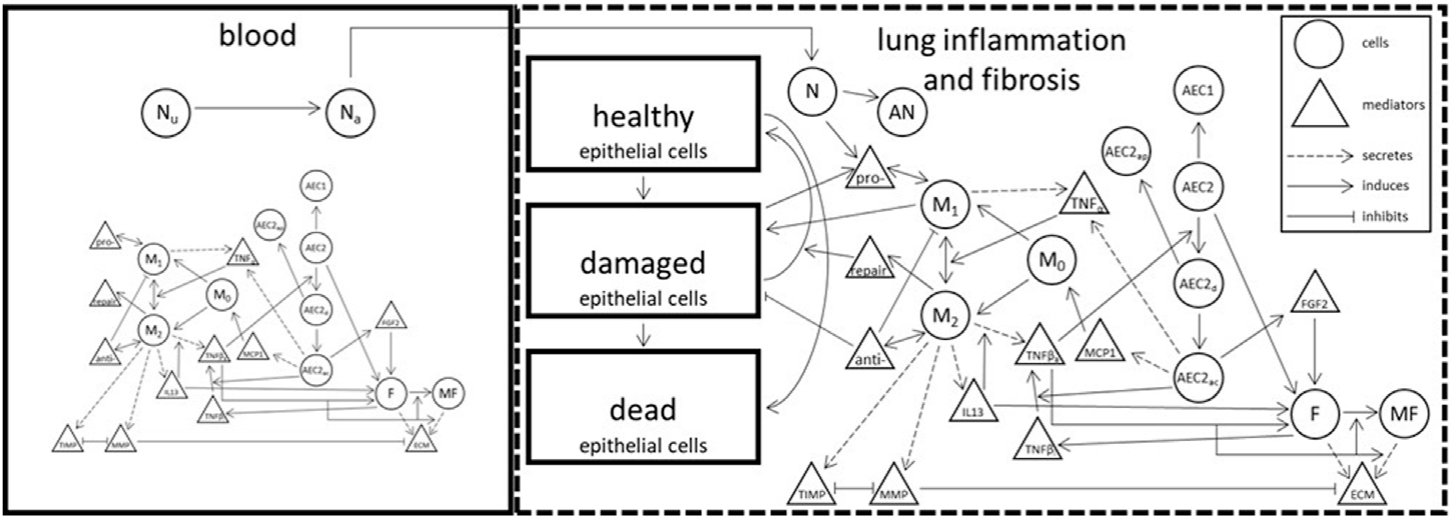
Abstract representation of model including all interactions [modified from (Cogo et al., 2007; Minucci et al., 2020).

At the lowest level of model granularity, compartments and their associated cellular-molecular interactions and distributions describing inflammation and fibrosis present in BPD (in both blood and lung tissue) are described. Figure 4 represents an idealized schematic from which the lowest level of model granularity is defined. Elements of the model consistent with the 3 goals described above are being codified and challenged by both preclinical and clinical SMEs. The working group plans to provide these early efforts to the broader BPD stakeholder community consistent with an Open Science framework. This will likely involve the creation of a secure Git Hub environment from which others can contribute in the future.

## Discussion

This is the first effort to review the landscape of BPD data, models, and other resources that could facilitate a strategy for the development of a family of quantitative models of BPD. While much of the emphasis is on RWD sources, the effort also includes the consolidation of preclinical data sources from relevant *in vitro* and animal experiments that would represent an anchor for mechanistic models including a QSP model. Complimentary models focused on the clinical value of current disease management practices more reliant on RWD are also a significant part of this effort. While interest is high for investigating the utility of RWD and real-world evidence (RWE) to inform drug development, we cannot assume that such data will fulfill its potential in all cases. A critical step in the context of use (COU) process is the definition of requirements and expectations regarding the performance of tools brought to regulators to support decision making. The ability of RWD sources and RWE derived from such sources to support such tools and the corresponding COU remains a work in progress reliant on the critical evaluation of data quality. Likewise, models constructed from or validated by such data must be of adequate quality to meet “fit-for-purpose” requirements as well. While there may be reasons to relax such requirements in situations where data is sparse and difficult to obtain, such decisions must be risk-based with adequate and well-informed justification from a diverse group of stakeholders.

Many knowledge gaps exist for BPD. While some may be addressed by accumulated and high-quality data, others will require more targeted investigation with attention to biomarkers both established and exploratory. In addition to the data, a multidisciplinary team of quantitative and clinical scientists must continue to challenge what has been evaluated thus far (preclinically and clinically), proposing experiments and analyses which help better define the disease progression as week as identify treatment options including a variety of modalities and disease progression in the context of distinct clinical phenotypes. Quantitative models serve the purpose of informing such prospective investigations based on scenario testing that evaluates and designs sampling times and frequency and sample size considerations (Mould, 2007; Cook and Bies, 2016). A disease progression model can also describe patient phenotypes and inform enrollment criteria as well as the timing of proposed interventions and treatments relative to the current standard of care (Cook and Bies, 2016; Gruneau et al., 2021). Hence, they have tremendous value for both the sponsors of such proposed interventions and regulators who must evaluate their safety and efficacy.

An important component of the disease progression model is the availability of credible longitudinal data in each patient. Such data would in theory capture the natural history of the disease and discriminate patient disease trajectories while examining response to treatment. Some examples of measure lab tests of clinical interest that could facilitate tracking of disease progression are summarized in Figure 2. In this respect the availability of RWD in BPD patients is theoretically of great value. Part of the challenge herein is to evaluate utility of the RWD based both on its quality, credibility and clinical information value and not assume that it is useful for this purpose based on its availability in the target population. Much of these data are still based on an opportunistic sampling approach given the fragility of the population. A plan to propose a DPM framework is still useful to identify both data and knowledge gaps as well as propose prospective study designs potentially incorporating more informative markers of disease progression at appropriate sampling times (Cook and Bies, 2016).

A key determinant for the overall success of this effort is the commitment for data sharing, collaboration, and transparency. The INC community is extremely knowledgeable and committed to the cause but relies on an extended group of stakeholders to deliver these high-quality data. It is also incumbent on pharmaceutical and academic researchers to promote the science, consider new biomarkers, use more innovative clinical trial designs, and remain unsatisfied with the status quo. Success for this effort demands this level of participation and investment. The proposed approach to consider RWD to guide models that inform BPD drug development is sound and rigorous. It will surely experience challenges and there can be no declared victories except continuing to fill gaps in our knowledge and understanding.

Our intention with this initial modeling effort is to build upon the data landscaping to produce a mechanistic QSP model as the starting point for a collaborative effort that eventually informs a BPD DPM. Measures of success (early and late) will be described further in an open-source format as the intention is to extend the initial FDA/INC-led effort to a broader community of BPD stakeholders including academic, regulatory, and industrial scientists.

## Data Availability

The original contributions presented in the study are included in the article/Supplementary Materials, further inquiries can be directed to the corresponding author.
