# Supplementary material for: Landscape analysis for a neonatal disease progression model of bronchopulmonary dysplasia: Leveraging clinical trial experience and real-world data
Source: Front Pharmacol. 2022 Oct 12;13:988974. doi: 10.3389/fphar.2022.988974 (PMC9597633; doi:10.3389/fphar.2022.988974)
Supplement: Supplementary file 1 [file DataSheet1.docx]

RWD available to construct BPD Disease Progression Model from committed sources to the recent INC / C-Path Grant with FDA

| **Data Type** | **Contributing Organization** | **Time Frame (Years)** | **Sample Size** | **Study Title /  EHR Vendor** | **ClinicalTrials.Gov ID** |
| --- | --- | --- | --- | --- | --- |
| Clinical Trial | Tufts Medical Center | 2013-2017 | 88 | Efficacy of Recombinant Human Clara Cell 10 Protein (rhCC10) Administered to Premature Neonates with Respiratory Distress Syndrome | NCT01941745 |
| Clinical Trial | Chiesi | 2017-2020 | 166 | A Study to Investigate The Safety, Tolerability And Efficacy Of Nebulised Curosurf In Preterm Neonates With Respiratory Distress Syndrome (RDS) | NCT03235986 |
| Clinical Trial | NICHD / NRN | 2005-2009 | 1316 | Surfactant Positive Airway Pressure and Pulse Oximetry Trial (SUPPORT) | NCT00233324 |
| Observational Studies | Tufts Medical Center | 2010-2012 | 86 | Improving Bronchopulmonary Dysplasia (BPD) Predictors and Outcomes for Clinical Trials |  |
| Observational Studies | UK NNRD |  |  |  |  |
| Observational Studies | Japan NRN |  |  |  |  |
| EHR | Advocate Children's Hospital - Oak Lawn |  | 1869 / year | Cerner |  |
| EHR | Advocate Children's Hospital – Park Ridge |  | 1541 / year | Epic |  |
| EHR | Ann and Lurie Children’s Hospital |  | TBD | TBD |  |
| EHR | Children’s National Hospital |  | TBD | TBD |  |
| EHR | Cleveland Clinic Children’s |  | 11500 / year | Epic |  |
| EHR | Johns Hopkins University - Baltimore |  | 850 / year | Epic |  |
| EHR | Le Bonheur Children's Hospital |  | ~ 600 / year | Cerner |  |
| EHR | Lucile Packard Children's Hospital |  | 900 / year | Epic |  |
| EHR | University of Utah |  | TBD | TBD |  |
| EHR | Medstar Medical Center |  | TBD | TBD |  |
| EHR | Nationwide Children’s Hospital |  | TBD | Epic |  |
| EHR | Nemours Children's Specialty Care, Delaware |  | 1197 / year | Epic |  |
| EHR | Nemours Children's Specialty Care, Jacksonville, FL |  | 870 / year | Cerner |  |
| EHR | Orlando Health-Arnold Palmer Children's Hospital |  | 1400 / year | Allscripts/Sunrise |  |
| EHR | University of Colorado |  | ~ 1050 / year | Epic |  |
| EHR | Prisma Health Children's Hospital |  | TBD | TBD |  |
| EHR | Rhode Island Hospital/Hasbro Children’s |  | TBD | TBD |  |
| EHR | Riley Children's Hospital |  | TBD | TBD |  |
| EHR | Rutgers-Robert Wood Johnson Medical School |  | 20 / year | SCM |  |
| EHR | Texas Children’s |  | TBD | TBD |  |
| EHR | Baystate Children’s Hospital |  | 70 / year | Cerner |  |
| EHR | University of Louisville-Norton Children’s Hospital |  | 1300 / year | Epic |  |
| EHR | University of New Mexico Health Sciences Center |  | TBD | TBD |  |
| EHR | University of Texas Health Sciences |  | TBD | TBD |  |
| EHR | VCU Children's Hospital |  | TBD | TBD |  |
| EHR | West Virginia University Children's Hospital |  | TBD | TBD |  |
| EHR | Yale New Haven Hospital |  | TBD | TBD |  |
| EHR | Children’s Hospital of Philadelphia |  | TBD | TBD |  |
| EHR | Hennepin County Medical Center |  | TBD | TBD |  |
